# Adult scoliosis can be reduced through specific SEAS exercises: a case report

**DOI:** 10.1186/1748-7161-3-20

**Published:** 2008-12-16

**Authors:** Alessandra Negrini, Silvana Parzini, Maria Gabriella Negrini, Michele Romano, Salvatore Atanasio, Fabio Zaina, Stefano Negrini

**Affiliations:** 1 Centro Negrini – ISICO (Italian Scientific Spine Institute), Corso Pavia 37, 27029 Vigevano (PV), Italy; 2 Centro Fisioterapia Negrini, Via Madonna degli Angeli 20, 27029 Vigevano (PV), Italy; 3 ISICO (Italian Scientific Spine Institute), Via Roberto Bellarmino 13/1, 20121 Milan, Italy

## Abstract

**Background:**

It has been known since many years that scoliosis can continue to progress after skeletal maturity: the rate of progression has shown to be linear, and it can be used to establish an individual prognosis. Once there is progression there is an indication for treatment: usually it is proposed a surgical one. There are very few papers on an alternative rehabilitation approach; since many years we propose specific SEAS exercises and the aim of this study is to present one case report on this approach.

**Case presentation:**

All radiographs have been measured blindly twice using the same protractor by one expert physician whose repeatability error proved to be < 3° Cobb; the average measurement has been used. In this case a 25 years old female scoliosis patient, previously treated from 14 (Risser 1) to 19 years of age with a decrease of the curve from 46° to 37°, showed a progression of 10° Cobb in 6 years. The patient has then been treated with SEAS exercises only, and in one year progression has been reverted from 47° to 28.5°.

**Conclusion:**

A scoliosis curve is made of different components: the structural bony and ligamentous components, and a postural one that counts up to 9° in children, while it has not been quantified in adults. This case shows that when adult scoliosis aggravates it is possible to intervene with specific exercises (SEAS) not just to get stability, but to recover last years collapse. The reduction of scoliotic curve through rehabilitation presumably does not indicate a reduction of the bone deformity, but rely on a recovery of the upright postural collapse. This reduction can decrease the chronic asymmetric load on the spine and, in the long run, reduce the risks of progression.

## Background

It has been known since 1969 that scoliosis can continue to progress during adulthood after skeletal maturity [1]. Its evolution is slow and insidious and involves both the anatomic and the functional aspect of the curve (development or worsening of painful spinal or radicular symptoms and/or disequilibrium) [2]. Once an asymmetric load or degeneration occurs, the pathomorphology and pathomechanism in adult scoliosis is quite predictable. Asymmetric degeneration leads to increased asymmetric load and therefore to a progression of the degeneration and deformity [3].

68% of cases of scoliosis progress, particularly curves measuring more than 30 Cobb degrees at skeletal maturity, regardless of the curve pattern [4,5]. Marty-Poumarat [6] showed that the rate of progression in adult scoliosis is linear, and it can be used to establish an individual prognosis.

Three types of treatment are available: rehabilitation, orthopaedic treatment and surgery. In literature, the prevailing treatment is surgery [3]. Somebody proposes also rehabilitation, with good short-term results [7-10]. Nevertheless, today we need more data on adult scoliosis natural history and rehabilitation treatment to understand how the deformity behaves and how it can be managed conservatively.

Since many years we propose specific exercises for adult scoliosis [11,12] according to the SEAS protocol [13-17], and our experience seems to sustain this possible treatment, even if we still lack data on its effectiveness.

This case report shows astonishing results obtained through SEAS exercises only.

## Methods

To reduce the possibility of measurement errors, all radiographs have been measured twice blindly (inserted in between other radiographs and not knowing the name of patients nor the stage of treatment) using the same protractor by one expert physician (SN): in a previous study his repeatability error proved to be less than 3° Cobb [18]. In this paper we report the average of these two measurements.

## Case presentation

**Figure 1:**
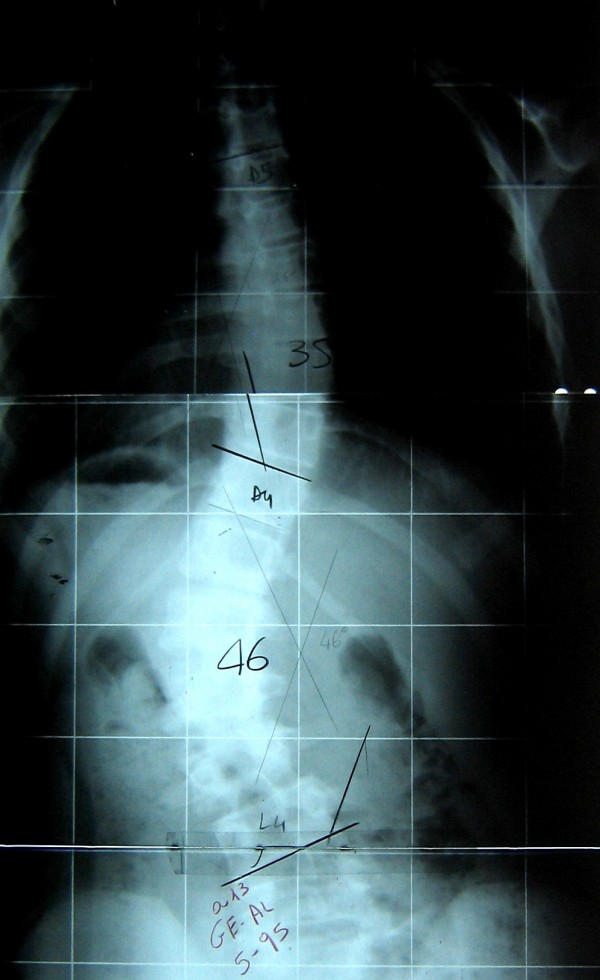
**Before adolescent treatment**. B.I. was 14 years old and Risser 1 when she discovered a right thoracic curve of 35° and a left lumbar of 46°.

**Figure 2:**
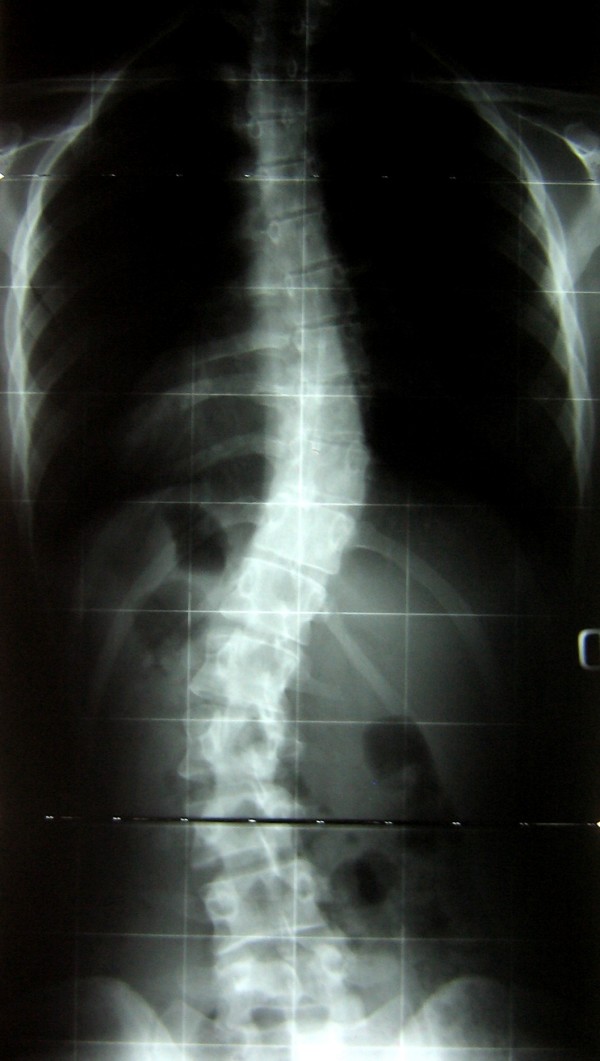
**After adolescent treatment**. At Risser 5 after 4 years of casts and braces curves were respectively 36° and 37°.

**Figure 3:**
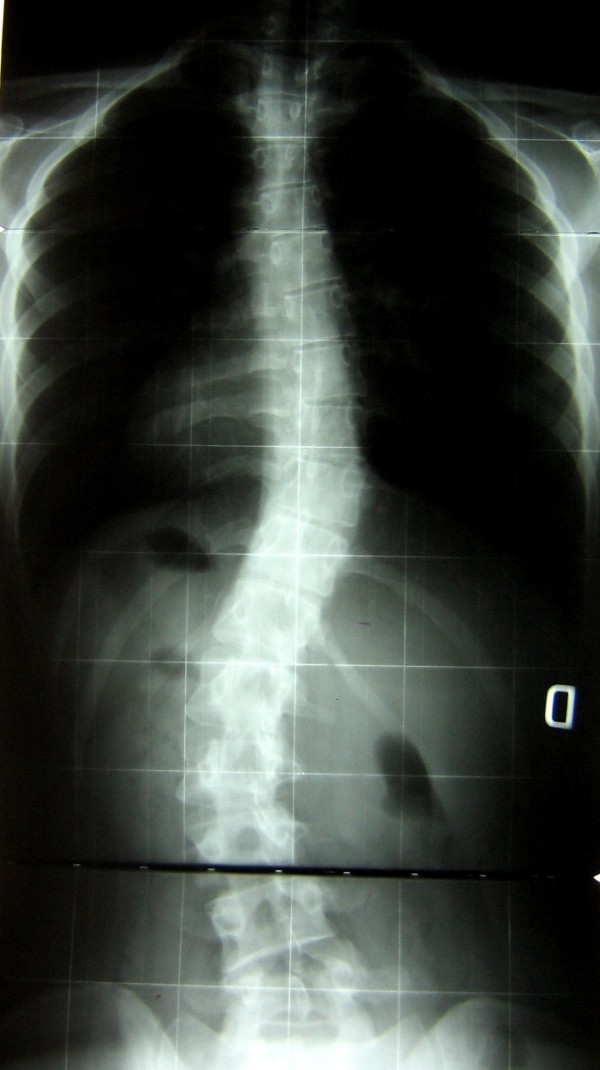
**At 1 year follow-up**. The 1 year follow-up showed stability with right thoracic 32.5° and left lumbar 38°.

**Figure 4:**
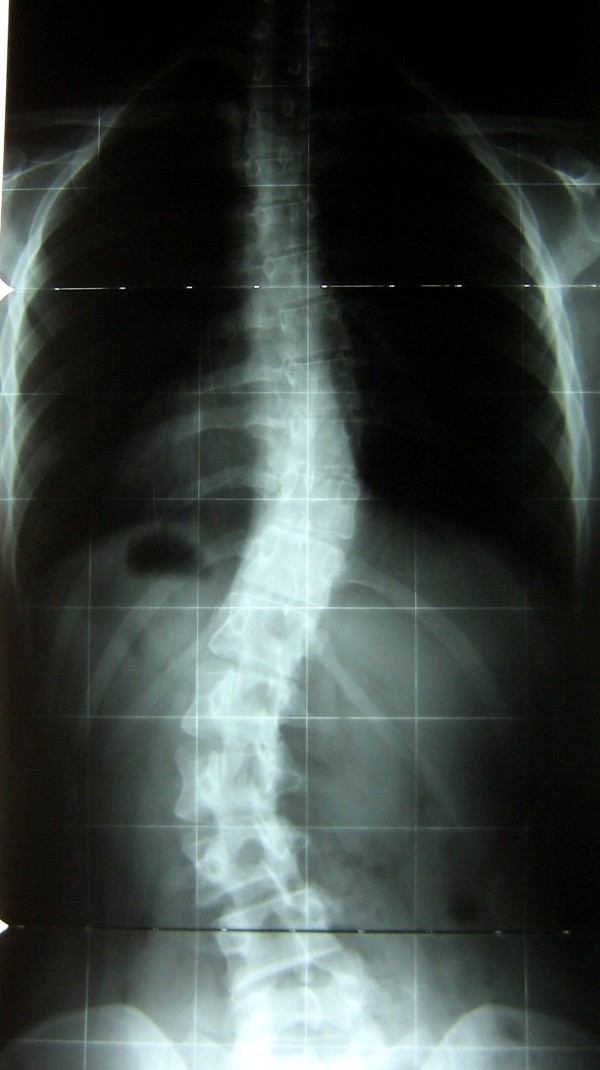
**At 5 year follow-up**. The 5 years follow-up showed a slight progression to right thoracic 31.5° and left lumbar 40°.

**Figure 5:**
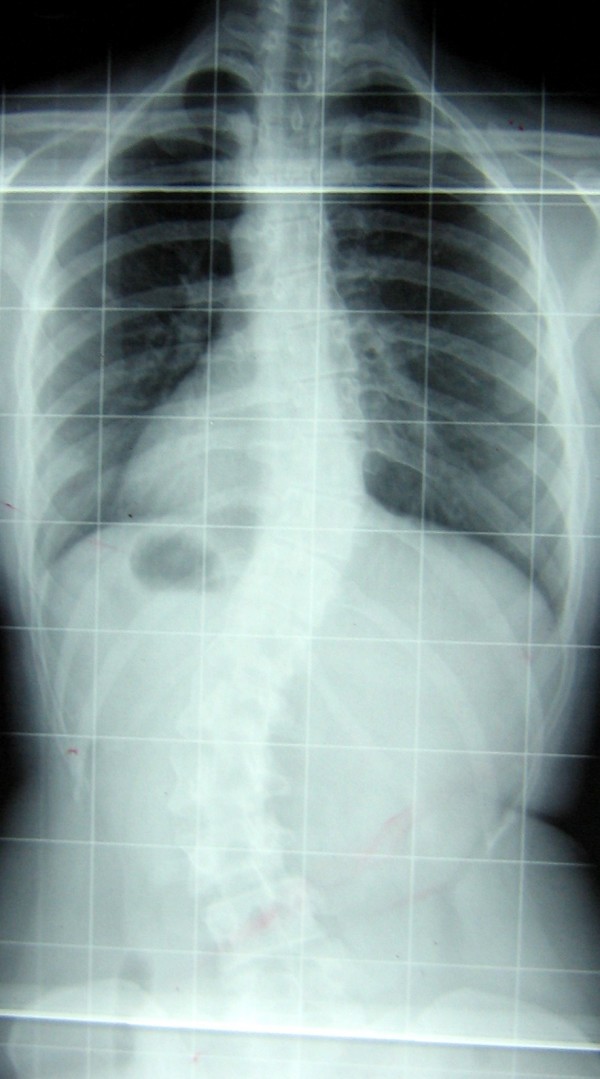
**Before adult specific SEAS exercises treatment**. After one year the progression was definite: right thoracic 35.5° and left lumbar 47° and SEAS specific exercises were started.

B.I. is a secretary. At the age of 14 (still Risser 1) her mother discovered that something was wrong with her back and she did her first x-rays showing a right thoracic (RT) curve of 35° Cobb, and a left lumbar (LL) of 46° Cobb (Figure 1). She has been treated during growth with Risser casts and braces with the final result when she was 19 years old of a RT curve of 36° and a LL of 37° (Figure 2). She was stable at 1 year follow-up (RT 32.5°, LL 38° – Figure 3), but the 5 years follow-up showed a slight progression (May 2005, RT 31.5°, LL 40° – Figure 4): in the meantime she had continued swimming, her preferred sporting activity. Being results inside the possible measurement error, it was only suggested to repeat the exam in one year, while continuing normally sport and other activities of day life. One year after, when she was 25, progression was definite (March 2006, RT 35.5°, LL 47° – Figure 5), and even increased. She had no back pain. SEAS exercises were started with the aim of avoiding surgery.

The patient has been treated with SEAS exercises only. SEAS is the acronym for "Scientific Exercises Approach to Scoliosis". SEAS originated about 30 years ago, and during this period it was continuously updated following acquisitions proposed by the scientific world. SEAS approach for adults with scoliosis is similar to SEAS approach for children. The goals at the neuromotor and biomechanical levels are the recovery of postural collapse, postural control and vertebral stability. Therapy includes at least two weekly exercise sessions lasting forty minutes each, that the patient can do at home or at the Center under the supervision of a qualified technician. The patient becomes aware of pathology consequences and recovery possibilities for postural collapse. She/he strengthens her/his muscles and stabilizes her/his spine in active self-correction, i.e., in the position of maximum postural collapse recovery. Postural integration is a key, including the neuromotor integration of correct postures and an ergonomic education program. SEAS exercises are aimed to improve patient's function.

In April 2006, when the program started, B.I. functional evaluation showed a bad, relaxed posture, with a thoraco-lumbar kyphosis. One leg Romberg test was inadequate, spinal range of motion (ROM) was high in all directions except extension, and the same was in all joints. Her upper and lower limbs muscular strength was fairly good, but her abdominal and back extensor muscles, her trunk antigravity muscles and gluteus muscles were weak. B.I. reported that while wearing casts and braces, she performed exercises only lying supine, and the only sport she played since she was a child was swimming.

**Figure 6:**
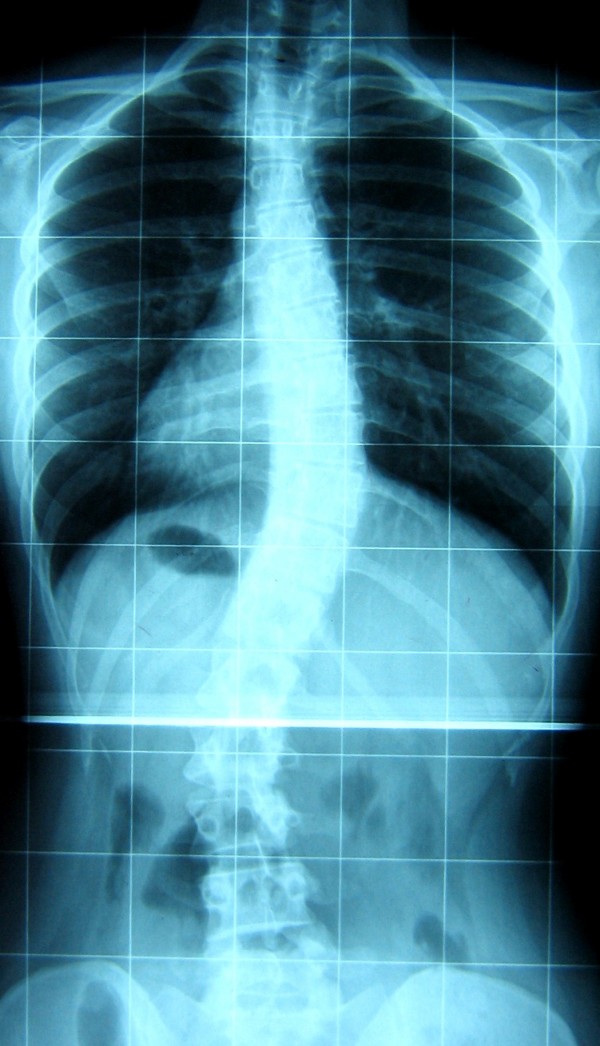
**After one year of adult specific SEAS exercises treatment**. After one year of SEAS exercises she had a scoliosis right thoracic 32° and left lumbar 28.5°.

B.I. started performing SEAS exercises 30 minutes every day at home, and came to the Center every two months to check and intensify her exercises. In one year B.I. recovered her posture, and according to radiographs, she was even better than at the end of bracing treatment (March 2007, RT 32°, LL 28.5° – Figure 6). In march 2007, functional evaluation showed a recovery of the thoraco-lumbar kyphosis, Romberg test was good, spine ROM was still high, and trunk extensor muscles, trunk antigravitary muscles and gluteus muscles very good.

## Discussion

**Figure 7:**
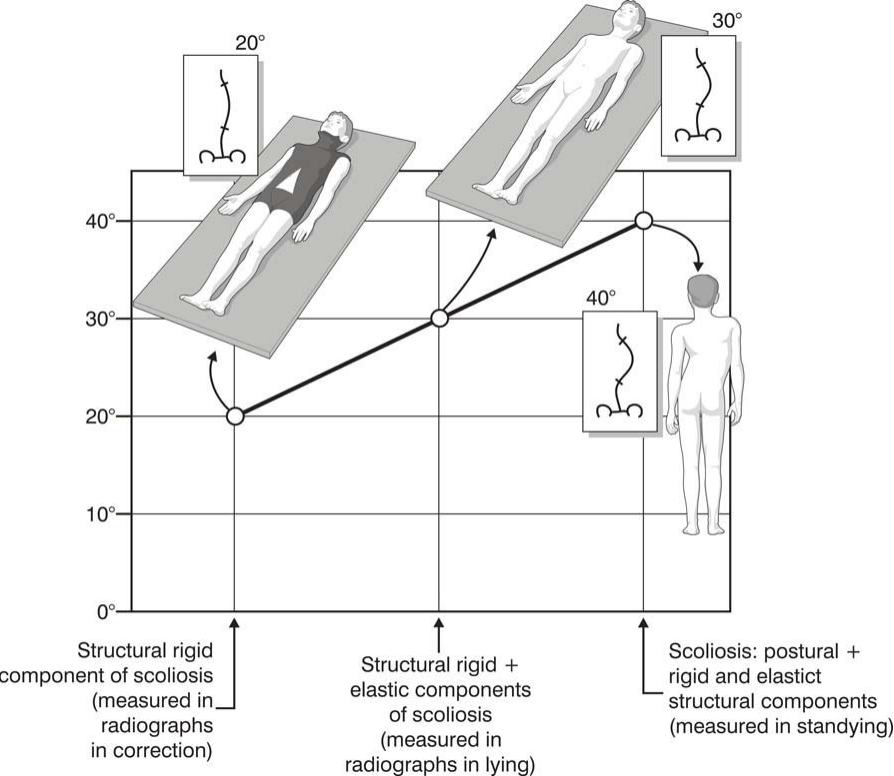
**The postural component of scoliosis **[19]. A scoliosis curve is made of many different components, including a postural one. Duval-Beaupére [19] described the case of three different radiographs: standing (SR), lying down (LR) and in correction e.g. using a cast (CR). The structural bony component can be measured with the CR; the structural ligamentous component comes from the difference between LR and CR; the postural component from the difference between SR and LR.

A scoliosis curve is made of many different components, including a postural one. Duval-Beaupére [19] described the case of three different radiographs: standing (SR), lying down (LR) and in correction e.g. using a cast (CR). The structural bony component can be measured with the CR; the structural ligamentous component comes from the difference between LR and CR; the postural component from the difference between SR and LR (Figure 7). In other words the CR gives the fixed deformity, while the classical SR gives the entire scoliosis, made up of the three components. The reduction of scoliotic curve through rehabilitation presumably does not indicate a reduction of the bone deformity, but rely on a recovery of the postural collapse, which is present in upright posture [11].

There is evidence that in adolescents, regardless of curve magnitude, the mean difference between a standing radiograph and a supine one is 9° Cobb [20], or 19% [21]. There are no data in the literature to indicate precisely what this difference is in adult scoliotic patients, even if it has been shown in scoliotics deformities with severe curves (mean Cobb angle 60 degrees) that performing an x-ray in different hours of the day [22] can give a measurement error due to worsening while the days goes on. This is another proof of the postural collapse. In adolescents, the curve decreases in mild scoliosis and in younger, less stiff patients, while an increase occur in older, more skeletally mature and heavier individuals [21]: accordingly, the postural collapse could be more important in adults than during growth.

While in adolescents exercises' aim is to reduce postural collapse and rehabilitate movement in this way allowing a better growth of the vertebrae [11,23], in adults the process to be controlled is bony deformation due to degeneration. Again, postural collapse can have a role, in the long term, of facilitating progression because of chronic asymmetric increase of compression. Obviously, also the increased quality of movement and the biomechanical changes of the spinal soft tissues could play a role in decreasing the risk of progression. [23,24]

This case opens the possibility that when adult scoliosis aggravates it is possible to intervene with specific exercises not just to get stability, but to recover last years collapse. We hypothesize that this reduction of Cobb degrees is due to a reduction of the postural collapse, that in turn can decrease the chronic asymmetric load on the spine and, in the long run, reduce the progression.

In this case report, we hypothesize that the worsening before the beginning of exercise therapy was due to an inadequacy of antigravitary trunk muscles due to all the years B.I. used casts and braces. During those years, she unfortunately never exercised to maintain or to restore these muscles. The only sport she played was swimming, which doesn't stress such muscles. With exercises, she could completely recover the worsening of the last five years.

Obviously exercises can lead to results other than stabilization of the curve, including pain, postural control, balance, strength, but in the indexed literature no data have been published on this topic. There are few works in literature showing it is possible improving the curvature in adult scoliosis with exercises, but no study has a long follow-up. The only case report with a long follow-up showed stability over time [25]. In our opinion, the recovery of the postural collapse we obtained in this case can possibly reduce the risk of future worsening, aside from the absolute decrease of the curves we had. In fact, the functional, cosmetic and psycho-social damages caused by scoliosis are directly proportional to curve magnitude [26,27], so any improvement over time must be considered a remarkable success in adult scoliosis therapy.

This case opens up a new perspective in the approach to adult scoliosis patients that appear to progressively worsen. Instead of a fatalistic "wait and see" approach, an active one could be envisioned and could help in some cases at least to postpone, and eventually avoid surgery. The advantage of maintaining spine mobility function and avoiding the risks of surgery is counterbalanced by the need of exercising regularly and being followed up carefully in a specialised Center by a rehabilitation team [28]. Spinal mobility allow a distribution of static and dynamic loads all over the spine, avoiding undue overload on single segments; it also permits to continue activities of daily life without any of the limitations that caution impose to fused patients. The final choice is obviously up to the patient [29,30], made aware of advantages and disadvantages of each option, understanding the difference between a risky one-shot treatment and a long-term, continuous one that requires collaboration and motivation, while in any case giving the possibility to go back to the other therapeutic option in case of failure. If other studies will confirm this case report, exercises could be considered and discussed with patients, allowing them to reach the best individual solution for their long term scoliosis management [30].

Another question opened up by this paper is: which kind of exercises can reduce adult scoliosis? We applied specific exercises (SEAS) that already showed to be useful in adolescent idiopathic scoliosis when compared to other exercises [17,31]. In the literature there are published results only on very short-term treatments, using Scoliosis Intensive Rehabilitation (SIR) [7], or manipulations [8,9]; there are no long-term answers on the possible efficacy in reducing the curve of specific exercises for adults, nor there are comparisons of different kind of exercises.

Finally, another issue: should we consider these changes to be real? In this paper all measurements have been made blindly twice by an expert physicians (SN), whose repeatability error has been previously evaluated to be less than 3° [18]. Obviously we must add to this value the repositioning error due to different radiographs in different days [32]. In any case, we used the average of the two measurements, so reducing even more the probability of error. All changes recorded were over the 5° classically considered in the literature; moreover, the radiographic variations have been accompanied by clinical significant changes (humps). Accordingly, we can be confident that all changes were real.

## Conclusion

This case report shows it is possible obtaining a significant improvement of scoliosis in adults with SEAS exercises. Marty-Poumarat showed that rate of progression in adult scoliosis is linear [6]. So, it is possible to establish an individual prognosis repeating x-rays every 4–5 years in adulthood. When x-rays detect a significant worsening, it is possible to recover and then to possibly stabilize scoliosis through SEAS exercises, thus avoiding the need of surgery.

## Competing interests

The authors declare that they have no competing interests.

## Authors' contributions

All authors made substantial contributions to conception, design and acquisition of data; they have been involved in drafting and revising the manuscript; they have given final approval of the version to be published.
